# Dynamic Properties of Water Confined in Graphene-Based Membrane: A Classical Molecular Dynamics Simulation Study

**DOI:** 10.3390/membranes9120165

**Published:** 2019-12-04

**Authors:** One-Sun Lee

**Affiliations:** Qatar Environment and Energy Research Institute, Hamad Bin Khalifa University, Doha P.O. Box 5825, Qatar; olee@hbku.edu.qa; Tel.: +974-4454-7184

**Keywords:** graphene, molecular dynamics simulation, membrane, free energy, desalination

## Abstract

We performed molecular dynamics simulations of water molecules inside a hydrophobic membrane composed of stacked graphene sheets. By decreasing the density of water molecules inside the membrane, we observed that water molecules form a droplet through a hydrogen bond with each other in the hydrophobic environment that stacked graphene sheets create. We found that the water droplet translates as a whole body rather than a dissipate. The translational diffusion coefficient along the graphene surface increases as the number of water molecules in the droplet decreases, because the bigger water droplet has a stronger van der Waals interaction with the graphene surface that hampers the translational motion. We also observed a longer hydrogen bond lifetime as the density of water decreased, because the hydrophobic environment limits the libration motion of the water molecules. We also calculated the reorientational correlation time of the water molecules, and we found that the rotational motion of confined water inside the membrane is anisotropic and the reorientational correlation time of confined water is slower than that of bulk water. In addition, we employed steered molecular dynamics simulations for guiding the target molecule, and measured the free energy profile of water and ion penetration through the interstice between graphene sheets. The free energy profile of penetration revealed that the optimum interlayer distance for desalination is ~10 Å, where the minimum distance for water penetration is 7 Å. With a 7 Å interlayer distance between the graphene sheets, water molecules are stabilized inside the interlayer space because of the van der Waals interaction with the graphene sheets where sodium and chloride ions suffer from a 3–8 kcal/mol energy barrier for penetration. We believe that our simulation results would be a significant contribution for designing a new graphene-based membrane for desalination.

## 1. Introduction

The separation of salt ions from seawater using graphene-based membranes is an emerging desalination method [1,2,3]. According to previous simulation and theoretical works, graphene-based membranes could achieve water permeability up to 1000 times greater than that of commercial seawater membranes [4,5]. Therefore, there have been many experimental approaches for developing better graphene-based membranes for desalination with the support of computational simulations [6,7,8,9,10,11,12,13,14]. For example, Sint et al. showed that functionalized nanopores in a graphene monolayer could serve as ionic sieves of high selectivity and transparency [12]. Cohen-Tanigi and Grossman showed that nanosized pores in single-layer freestanding graphene can filter salt from water by using a molecular dynamics (MD) simulation [4]. The formation and melting of a hexagonal ice bilayer between graphene oxide sheets and their effect on water permeation was studied by Boukhvalov et al. [6]. However, most simulations of desalination use only one or a few sheets of graphene for modeling the membrane. With this minimal model, the information of the dynamic properties of water and ions is restricted, and this has proven to be a hindrance in understanding how these systems function since the properties of confined water in nanoscale pores or channels of membranes are expected to be different from those of bulk liquid.

**Figure 1:**
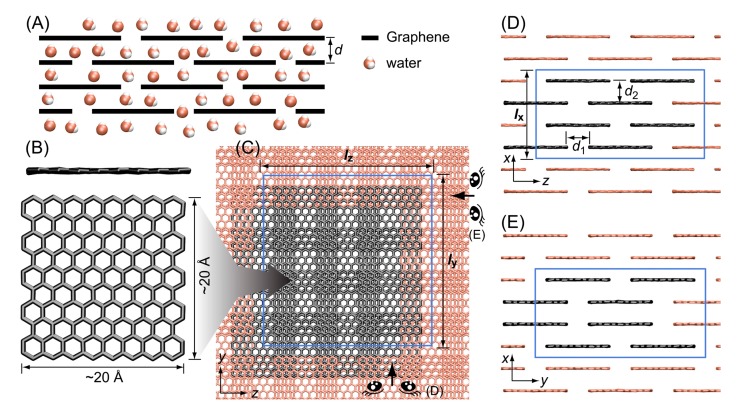
(**A**) Schematic representation of the penetration of water through the interlayer of stacked graphene sheets. (**B**) Snapshots of the side and top view of the graphene sheet used in the simulations. (**C**) Top view (x-y plane) of the stacked graphene sheets. The graphene sheets that belong to the main simulation box are shown in black, their periodic images are shown in red, while the periodic boundaries are represented by blue lines. Side views of (**D**) x-z plane and (**E**) y-z plane are shown with the periodic boundaries represented by blue lines. The directions of the views of (**D**) and (**E**) are indicated by the schematic eye symbols. The distances of the horizontal (*d*_1_) and vertical interlayers (*d*_2_) were set to the same value of *d* = 7 Å for all simulations.

Here, we developed a model membrane system based on the experimentally suggested [1,2,3] stacked graphene configuration (Figure 1A). To develop an in silico model of a graphene-based membrane, we configured an infinitely repetitive structure of stacked graphene sheets using the periodic boundary conditions (PBC) [15]. In PBC, when a molecule passes through one side of the main unit cell, it comes back on the opposite side, and each individual particle in the main simulation cell interacts with the closet image cell (Figure 1B–E). While keeping the same distance between the graphene sheets along all the directions over image cells, we could develop a membrane model composed of a repetitive structure of stacked graphene configurations. This membrane provides a hydrophobic environment, and we could interrogate the dynamic properties of water molecules inside this hydrophobic environment by performing an MD simulation. We found that water molecules self-assemble into a droplet through hydrogen bond networks in the hydrophobic environment when the density of water molecules is lower. We suggest that a bigger water droplet has a stronger van der Waals interaction with the graphene surface, and the stronger interaction hampers the translational motion of the droplet. Therefore, the translation diffusion coefficient of water increases as the size of the water droplet decreases. We also found that hydrogen bond lifetime increases as the size of water droplet decreases because the hydrophobic environment limits the libration motion of water molecules. We also developed another model membrane system to study the translation of water molecules and ions between the hydrophobic membranes and bulk water. With this second model membrane, we could measure the free energy profile of the penetration of water molecules and salt ions from bulk water to the hydrophobic membrane. The free energy profile of the penetration revealed that the optimum interlayer distance between graphene sheets for desalination is ~10 Å. We believe that our simulation results would be a significant contribution for designing a new graphene-based membrane for desalination.

## 2. Computational Details

### 2.1. Force Field Parameters

The energy function used in the simulations had the form

(1) $$\begin{array}{l} U\left(b,\theta ,\chi ,\varphi ,r_{ij}\right)=\sum_{bonds}K_{b}{\left(b-b_{0}\right)}^{2}+\sum_{angle}K_{\theta }{\left(\theta -\theta_{0}\right)}^{2}\\ +\sum_{dihedral}K_{\chi }\left(1+\cos\left(n\chi -\delta \right)\right)+\sum_{impropers}K_{imp}{\left(\varphi -\varphi_{0}\right)}^{2}\\ +\sum_{nonbond}\varepsilon_{ij}\left[{\left(\frac{R_{ij}^{\min}}{r_{ij}}\right)}^{12}-2{\left(\frac{R_{ij}^{\min}}{r_{ij}}\right)}^{6}\right]+\frac{q_{i}q_{j}}{\varepsilon_{0}r_{ij}} \end{array}$$

where *K_b_*, *K_θ_*, *K_χ_*, and *K_imp_* are bond, angle, dihedral angle, and improper dihedral angle force constants, respectively; *b*, *θ*, *χ*, and *φ* are bond length, angle, dihedral angle, and improper torsion angle, respectively. Lennard-Jones (LJ) 6–12 and Coulombic terms are used for the nonbonded interactions where *ε_ij_* is the LJ well depth, $R_{ij}^{\min}$ is the distance at the LJ minimum, *q*_i_ is the partial atomic charge, and *r*_ij_ is the distance between atoms *i* and *j*. The *ε_ij_* values were obtained via the geometric mean ($\varepsilon_{ij}=\sqrt{\varepsilon_{ii}\varepsilon_{jj}}$) and $R_{ij}^{\min}$ via the arithmetic mean ($R_{ij}^{\min}=\frac{R_{i}^{\min}+R_{j}^{\min}}{2}$). There have been various implicit and explicit solvation models used in MD simulations [16,17,18,19,20]. The implicit solvation model represents the solvent as a continuous medium that accommodates enthalpic and entropic contributions. Adapting different water models in the simulation affects the simulation results significantly. According to Lee et al., for example, changing the effective dielectric constant (*ε*_0_) from 1 to 10 lowers the intramolecular rotational barrier (thus weakening the molecular recognition) via the charge screening [20]. Meanwhile, explicit models adapt direct solvent molecules where the value of effective dielectric constant (*ε*_0_) is set to 1, and it is well known that many properties of bulk phase water including diffusion coefficients [21], surface tension [22], and isothermal compressibility [23] could be reproduced by adapting explicit water models. However, at the same time, we still suffer from reproducing the many properties of bulk water such as second virial coefficient, vapor pressure, and dielectric constant with the explicit water models [24]. With a non-polarizable explicit water model, for example, the dipole moment fluctuation of the confined water molecules is difficult to obtain so the reproduction of the dielectric constant is not sufficient to compare with experiments [25]. In our work, the extended simple point charge (SPC/E) explicit water model [21] was adapted for all simulations, and the force field parameters for water and graphene interaction developed by Wu and Aluru were adapted (*R*_CO_ = 3.86 Å, $\varepsilon_{CO}$ = 0.085 kcal/mol, *R*_CH_ = 3.02 Å, and $\varepsilon_{CH}$ = 0.038 kcal/mol) [26].

### 2.2. Molecular Dynamics Simulations

The system was equilibrated for 1000 steps using the conjugate gradient method, followed by a 10 ns MD simulation at 300 K with a canonical ensemble (constant number of particle, volume, and temperature (NVT)). During MD simulation, the position of each sp^2^ carbon atom of the graphene sheets was fixed with a harmonic constraint of 10 kcal/mol/Å^2^ to maintain the distance between the sheets. Pressure was maintained at 1 atm using the Langevin piston method with a piston period of 100 fs, a damping time constant of 50 fs, and a piston temperature of 300 K [27,28]. Full electrostatics were employed using the particle-mesh Ewald method with an 1 Å grid width [29]. Nonbonded interactions were calculated using a group-based cutoff with a switching function and updated every tenth time-step. Covalent bonds involving hydrogen were held rigid using the SHAKE algorithm [30], and a 1 fs time step was used. Atomic coordinates were saved every 1 ps for the trajectory analysis. The trajectories obtained from this simulation were used for calculating the translational diffusion coefficient of water in each system. All MD simulations were carried out using nanoscale molecular dynamics (NAMD) [31] and the graphics shown in this report were prepared using visual molecular dynamics (VMD) [32].

## 3. Results and Discussion

### 3.1. Stacked Graphene Membrane

**Figure 2:**
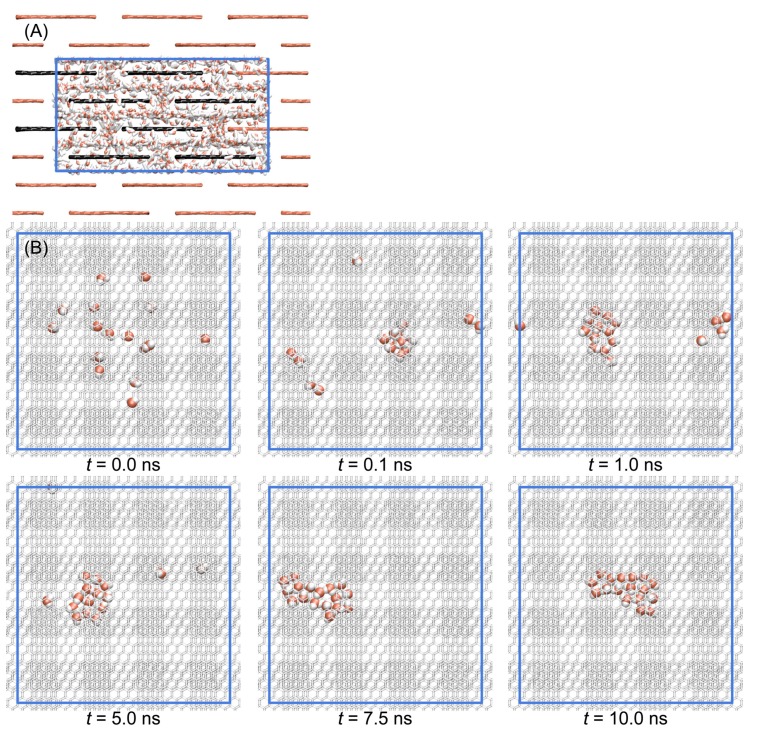
(**A**) A snapshot taken at *t* = 10 ns when the number of water molecules was 1698. The main image of the graphene sheets is in black while their periodic images are in red. Periodic images of water are not shown for clarity. (**B**) Snapshots of molecular dynamics (MD) simulations when the number of water molecules was 17. Blue lines are used for representing the periodic boundaries.

The stacked graphene membrane was composed of four layers of graphene sheets, and each layer was composed of four rectangular graphenes with the dimension of ~20 Å × ~20 Å (Figure 1B). The distance between sheets in the same layer (*d*_1_) and the distance between layers (*d*_2_) was set as 7 Å, which is the minimum distance water molecules can penetrate [33]. Since the dimension of the sheets and the distance between them were fixed, the dimension of the periodic simulation box was also determined: 28.0 Å × 53.2 Å × 53.8 Å (*l*_x_ × *l*_y_ × *l*_z_ in Figure 1C,D). To maintain the structure of the periodic membrane, we had to keep the same dimension size of each direction so we could adapt a NVT ensemble for MD simulations. To determine the appropriate number of water molecules in the fixed volume of the periodic box, we tested several 1 ns MD simulations with different numbers of water molecules using a isothermal-isobaric ensemble (constant number of particle, pressure, and temperature (NPT)) and found that 1698 SPC/E water molecules are appropriate for the desired volume (80,140.5 Å^3^ = 28.0 Å × 53.2 Å × 53.8 Å) of the membrane. The volume fluctuation of 1698 SPC/E water molecules during 1 ns MD simulation with NPT ensemble was less than 1% of the desired volume. Then, we also performed MD simulations with 850 (≈12×1698), 17 (≈1100×1698), and 3 (the smallest number of waters possible for observing hydrogen bond networks) water molecules with the same dimensions to investigate the effects of the density of water inside membranes. To maintain the structure and the periodicity of the membrane, we also had to keep the position of the graphene sheets the same. Therefore, all of the sp^2^ carbon atoms of graphene were fixed at the starting position with a harmonic constraint of 10 kcal/mol/Å^2^. We monitored the structural fluctuations of each graphene sheet to ensure the desired interlayer distance between the membranes was maintained during the MD simulations, and we found that each sheet maintained the planar structure, and the distance between sheets was maintained well at the desired value (See Figure 2A). We also found that water molecules formed droplets when the density was lower. When the number of water molecules was 17, water molecules formed a droplet within 1 ns and translated together (Figure 2B). We also observed that only a single water layer was formed between neighboring graphene sheets. This is consistent with the previous work by Martí et al. [33]. They reported that water molecules form a single layer when the interlayer distance between graphene sheets is 6.5–7 Å.

### 3.2. Diffusion of Water

The self-diffusion coefficient *D_i_* for species *i* was obtained by the Einstein relation equation [15,34],

(2) $$D_{i}=\underset{t\to \infty }{\lim}\frac{\langle {\left[r\left(t\right)-r\left(t_{0}\right)\right]}^{2}\rangle }{2dt}$$

where < > is the time average, *d* the dimension of the system, and *r*(*t*) the position of species *i* at time *t*. The self-diffusion coefficient is calculated by the slope of the fitted line of the mean squared displacement (MSD) versus time plot. 

**Figure 3:**
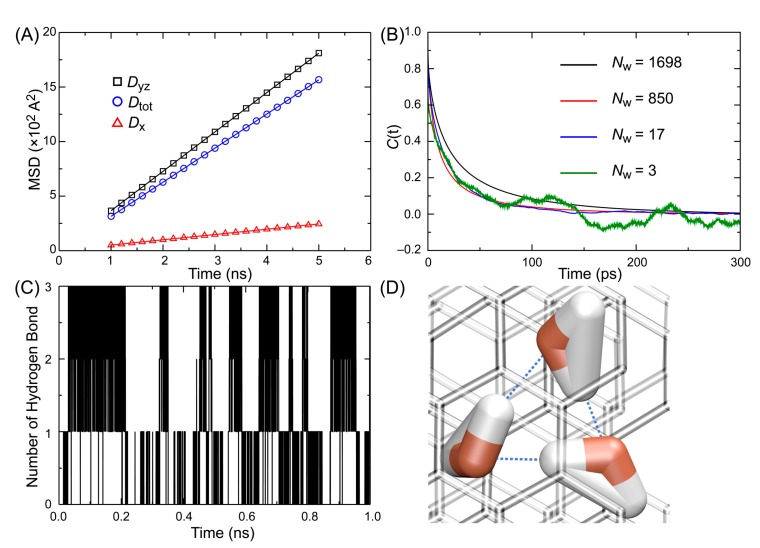
(**A**) The plots of mean squared displacement (MSD) versus time and their fitted lines of water molecules when the number of water molecules is 1698. (**B**) The autocorrelation function of hydrogen bond lifetime. (**C**) The fluctuation of the number of hydrogen bonds when the number of water molecules is three in the stacked graphene membrane. (**D**) A snapshot of a three-water molecule network where the water molecules interact by three hydrogen bonds to each other. Hydrogen bonds are indicated by the black dotted lines.

According to previous studies, the diffusion behavior of confined water is significantly different from that of bulk water [33,35,36]. For example, Martí et al. reported that when water molecules are inside carbon nanotubes (CNTs), the longitudinal diffusion coefficient of water along the axis of CNTs is larger than the diffusion coefficient along the normal to the axis of CNTs [33]. They also found that the overall diffusion coefficient (*D*_tot_) is approximately equal to the average of the coefficients in two directions, and *D*_tot_ is slightly larger than that of bulk water. We also measured the diffusion coefficient along the graphene plane (*D*_yz_) and the normal to the graphene plane (*D*_x_). The diffusion coefficient of water molecules in each system is shown in Table 1, and we found that *D*_tot_ increased as the density of water decreased. We also found that *D*_x_ was significantly smaller than *D*_tot_ when *N*_w_ = 1698 (See Figure 3A), and it was almost negligibly small when *N*_w_ = 850 or 17. It seems that water molecules form a droplet which is adsorbed on the surface of graphene when *N*_w_ = 850 or 17. The evaporation of water and translational movement along the *x*-axis is restricted because the van der Waals interactions between the water droplet and the graphene surface in addition to the hydrogen bond between water molecules. When *N*_w_ = 3, however, the values of *D*_yz_ and *D*_x_ were comparable, and they were larger than that of bulk water because the van der Waals interaction between the graphene surface, and the cluster of three water molecules was not strong enough to restrict the desorption of water. This is comparable with the report by Striolo et al. [37]. They performed MD simulations of the confined water in a CNT, and found that the diffusion speed of the smaller water cluster is faster than that of the bigger one along the axis of the CNT.

### 3.3. Reorientation Correlation Time

Many theoretical and experimental studies suggested that water molecules near surfaces would form an ordered structure and their motions would be restricted compared to the bulk phase. Recently, Fumagalli et al. [38] showed that confined water molecules between atomically flat surfaces have restricted rotational motions and their out-of-plane dielectric constant is ~2 because of the small polarization of water. Therefore, we could expect a slower rotational motion of the confined water molecules in the stacked graphene membrane. So, we characterized the rotational diffusion of the confined water where *N*_w_ = 1698 and *d* = 7 Å (See Table 2). The molecular reorientational correlation times were obtained from the integration of the molecular reorientational correlation function of each water molecule:

(3) $$\tau_{l}==\int_{0}^{\infty }C_{l}\left(t\right)dt=\int_{0}^{\infty }\langle P_{l}\left(\hat{u}\left(0\right)\cdot \hat{u}\left(t\right)\right)\rangle dt$$

where *P_l_*(x) is the *l*th order of Legendre polynomial and *û*(*t*) represents the unit vector bound to the water molecule at time *t*. The reorientational correlation times *τ*_1_ and *τ*_2_ have been computed for four different vectors: the OH-bond, the molecular dipole (*µ* or *z*-axis), HH-vector (*x* axis), and the vector perpendicular to the molecular plane (⊥ or *y*-axis). We found that the reorientational correlation time of confined water along each vector was slower than that of bulk water, as we expected. We also found that the rotational motion of a confined water molecule was anisotropic [39].

### 3.4. Hydrogen Bond Lifetime

Since the typical value of a hydrogen bond lifetime is ~1 ps, we needed to sample the MD trajectory with a higher sampling frequency. Therefore, we performed an additional 1 ns MD simulation for each system, and atomic coordinates were saved every 0.1 ps during this period. Using these samples, the lifetime of the hydrogen bond (*τ*_HB_) of water was obtained by introducing the method proposed by Chandler and Luzar [46,47,48]. The hydrogen bond correlation function *c*(*t*) is defined as

(4) $$c\left(t\right)=\frac{\langle h\left(0\right)h\left(t\right)\rangle }{\langle h\rangle }$$

where *h*(*t*) = 1 when the given pair of donor and acceptor molecules forms hydrogen bonds and 0 otherwise, and <*h*> is the average number of hydrogen bonds [47].

(5) $$D:A\overset{k_{1}}{\underset{k_{-1}}{⇄}}D\cdots A$$

Chandler and Luzar adapted a kinetic model for describing the hydrogen bond formation and breaking (Equation (5)) where *D*:*A* represents when the hydrogen bond is on and *D*⋯*A* represents when the hydrogen bond is off. For long-time *t*, the reactive flux is [46]:

(6) $$k\left(t\right)=-\frac{dc\left(t\right)}{dt}=-\frac{\langle \dot{h}\left(0\right)\left[1-h\left(t\right)\right]\rangle }{\langle h\rangle }\approx k_{1}e^{-k_{1}t}$$

With the least square fit method, the hydrogen bond lifetime (*τ*_HB_) can be obtained as *τ*_HB_ = 1/*k*_1_. Geometric criteria are used to determine the existing hydrogen bonds: 3.5 Å cutoff of the oxygen-oxygen separation, 2.45 Å cutoff of oxygen (acceptor) and hydrogen (donor) separation, and 30° of the angle between the oxygen–oxygen vector and the covalent OH-bond [49].

The autocorrelation functions of the hydrogen bond lifetime with different densities of water molecules are shown in Figure 3B, and the hydrogen bond lifetime is listed in Table 3. When the number of water molecules was 3, *τ*_HB_ showed the longest hydrogen bond lifetime. This agrees with the previous reports by Chandler and Luzar [46,48]. Libration motion is the main reason of the breaking of hydrogen bonds, and because of the hydrophobic environment of graphene, water molecules tend to form a droplet and avoid libration. As shown in Figure 3, 3 water molecules formed hydrogen bonds with each other during the whole simulation.

### 3.5. Potential of Mean Force (PMF)

**Figure 4:**
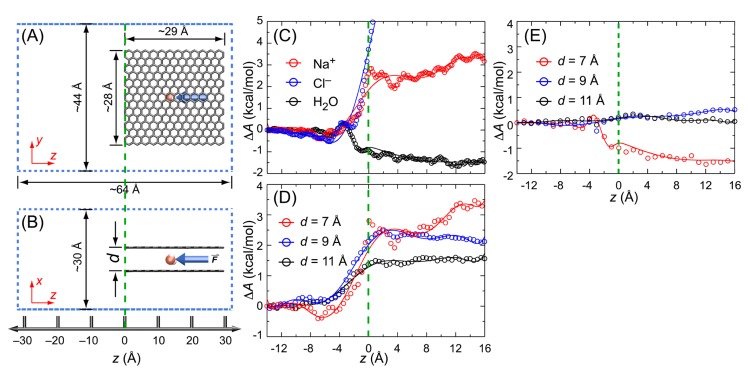
Schematic view of the system used for calculating the free energy profile of the translation of water and ions through the interlayer between graphene sheets (29 Å × 28 Å). The number of water molecules was 2372 and the number of sodium and chloride ions were 27 each. Water and ions are not shown for clarity. (**A**) The top (*y-z* plane) and (**B**) side (*x-z* plane) views of the system. The water molecule (or ion) is pulled from *z* = 16 Å to *z* = −14 Å for calculating the free energy profile. Dotted lines are used for representing the periodic boundaries. (**C**) The free energy profile of the penetration of water (or ions) between the two graphene sheets. The coordinate of the entrance of the space between two graphene sheets is set as *z* = 0, which is indicated by the green dotted line. (**D**) The free energy profile of the penetration of sodium ions between graphene sheets when the interlayer distance is 7, 9, or 11 Å. (**E**) The free energy profile of the penetration of a water molecule between graphene sheets when the interlayer distance is 7, 9, and 11 Å.

To investigate the free energy profile of the penetration of water molecules and ions between graphene sheets, we built a system composed of two graphene sheets in salt water where the concentration of sodium and chloride ions was ~0.5 m, that is, approximately the salt concentration of seawater. Schematic views of the system are shown in Figure 4A,B. The Kirkwood–Buff force field parameters were used for the simulation of sodium and chloride ions (*R*_Na-Na_ = 2.75 Å, $\varepsilon_{Na-Na}$ = 0.077 kcal/mol, *R*_Cl-Cl_ = 4.94 Å, and $\varepsilon_{Cl-Cl}$ = 0.112 kcal/mol) [54,55]. We calculated the free energy profile of the penetration of water molecules and ions through the interlayer between graphene sheets using a steered molecular dynamics (SMD) simulation [56]. During the SMD simulation, the position of the two graphene sheets was fixed by applying a harmonic constraint, while one water molecule (or an ion) was pulled with a constant velocity of 2.0 Å/ns. A harmonic constraint with a spring constant of 100 kcal/mol/Å^2^ was used for pulling one water molecule (or an ion), and the total length of the pulling reaction coordinate was 30.0 Å. We divided the reaction coordinate into three consecutive sections with each section length being 10.0 Å. At each section, the system was equilibrated for 1 ns while constraining the position of two sheets and a water molecule (or an ion), and SMD simulation data were collected during another 5 ns. Eight independent simulations were performed at each section for constructing the PMF.

The construction of the PMF from SMD simulations is based on the Jarzynski equality equation.

(7) $$\Delta A=-\beta^{-1}\ln\langle \exp\left[-\beta W\right]\rangle$$

where ∆*A* is a free energy difference, *β* is the product of Boltzmann factor and temperature, and *W* is the non-equilibrium work obtained from the SMD simulation. The non-equilibrium work done by the pulling force can be obtained using the following:

(8) $$W=-kv\int_{0}^{t}dt^{\prime }\left[x\left(t^{\prime }\right)-x_{0}-vt^{\prime }\right]$$

where *k* and *v* are the force constant and velocity of pulling, respectively, and *x*(*t*′) and *x*_0_ are the reaction coordinate at *t*′ in the simulation and the initial position of the center of mass of the pulled water molecule (or ion), respectively. We adapted the second order cumulant expansion equation for calculating Equation (7).

(9) $$\Delta A=\langle W\rangle -\frac{\beta }{2}\left[\langle W^{2}\rangle -{\langle W\rangle }^{2}\right]$$

The calculated free energy profiles are shown in Figure 4C. When water molecules stay in the interlayer space (*z* > 0), the hydrogen bond interaction probability is lower than the bulk state (*z* < 0) because only one layer of water cluster is formed and the hydrogen bond network is formed only in two-dimensions when the interlayer distance is ~7 Å. Because of the van der Waals interaction, however, water molecules are stabilized by ~1.5 kcal/mol when staying between graphene sheets compared to the bulk state. As we observed in the MD simulation, water molecules tended to form a droplet rather than disperse individually inside the interlayer space when *N*_w_ = 850, 17, or 3 because of the hydrogen bonds with each other. A bigger water droplet has a higher van der Waals interaction energy with the graphene sheets, and the higher interaction energy hampers the diffusion of the water droplet. In addition, the energy barrier for water inside the interlayer space is smooth, and this explains the fast translational diffusion of water when the density of water is lower. Sodium ions are destabilized by 3 kcal/mol when penetrating the space between graphene sheets because the three-dimensional water shell around the sodium ion has to be rearranged to a two-dimensional water shell. The same reason also applies to chloride ions where the free energy changes significantly even at the entrance of the interlayer space between two graphene sheets. The free energy barrier for chloride ion penetration is ~8 kcal/mol. We also obtained the free energy profile of sodium ions by varying the distance between graphene sheets (*d* = 7, 9, and 11 Å in Figure 4D). As expected, the free energy barrier of sodium ion penetration decreased as the interlayer distance increased. We also calculated the free energy profile of the penetration of water molecules at different interlayer distances (Figure 4E). When *d* = 9 or 11 Å, we observed that the stabilization of water by the van der Waals energy with graphene sheets was removed, and the free energy profile was almost flat along the translation coordinate. Therefore, the free energy difference between water and sodium ion for penetration is ~4 kcal/mol (*d* = 7 Å), ~2.5 kcal/mol (*d* = 9 Å), and ~1.5 kcal/mol (*d* = 11 Å). By considering the Boltzmann distribution, the sodium ion penetration ratio between *d* = 11 Å interlayer space and *d* = 7 Å interlayer space (*P*_d = 11_/*P*_d = 7_ ≅ exp(4/0.6)/exp(1.5/0.6)) is ~70 (RT ≅ 0.6 kcal/mol at 300 K). However, there are two factors that govern the efficiency of desalination membranes: the permeability of water and the salt rejection. Since the energy barrier of water is negligible when *d* > 9 Å, a graphene-based membrane with an interlayer distance larger than 9 Å is desirable considering the water permeability. In addition, we can expect that the sodium ion penetration ratio between membranes of *d* = 9 Å and *d* = 11 Å is *P*_d = 11_/*P*_d = 9_ = ~5. Therefore, we suggest that the optimum interlayer distance between graphene sheets for desalination is 9–11 Å. Based on the free energy profile calculations, we conclude that stacked graphene-based membranes have a potential application for desalination.

In summary, to explore the desalination applicability of stacked graphene-based membranes, we performed MD simulations of water molecules inside the bare graphene membrane where the interlayer distance between sheets was fixed at 7 Å, which is the minimum distance for water penetration. We also changed the density of the water inside the membrane and observed the density-dependent dynamic properties of water molecules. We found that water molecules formed a droplet inside the hydrophobic environment and water molecules translated together as a droplet rather than dispersed inside the membrane. The diffusion coefficient of water along the graphene surface was higher as the density of water decreased, because bigger droplets have a stronger van der Waals interaction with the hydrophobic surface of the membrane and that hampers the diffusion of the droplet. Using SMD simulations and the analysis with the Jarzynski equation, we also obtained the free energy profile of the penetration of water molecules and salt ions through the interlayer space between two stacked graphene sheets where the interlayer distance was 7, 9, or 11 Å. We found that the water permeation was desirable when *d* > 9 Å and the salt rejection energy barrier decreased as the interlayer distance increased. Based on the simulation results, we suggest that an interlayer distance of 9–11 Å is optimum for desalination when using a stacked graphene-based membrane.

## Figures and Tables

**Table 1:** The diffusion coefficient of water (Extended simple point charge model, SPC/E) inside a stacked graphene membrane with different numbers of water molecules. For comparison, we also listed the diffusion coefficient of bulk phase SPC/E water without the stacked graphene membrane (∞ *) as well as the previous experimental results (∞ **).

Number of Water Molecules (*N*_w_)	1698	850	17	3	∞ *	∞ **
*D*_tot_ (×10^−5^ cm^2^/sec)	0.60	1.19	1.20	5.44	2.39	2.3
*D*_yz_ (×10^−5^ cm^2^/sec)	0.78	1.77	1.82	5.41	-	-
*D*_x_ (×10^−5^ cm^2^/sec)	0.24	0.01	0.05	4.37	-	-
Density of water (H_2_O/Å^3^)	0.037	0.019	3.7 × 10^−4^	6.6 × 10^−7^	0.033	0.033

* Simulation; ** Experiment [21].

**Table 2:** Reorientation correlation time (ps) of water inside stacked graphene.

*d* (Å)	7	∞ *	∞ **
$\tau_{1}^{HH}$	6.66	4.28	-
$\tau_{1}^{\mu }$	13.86	4.71	4.76 [40]
$\tau_{1}^{⊥}$	5.44	2.88	-
$\tau_{1}^{OH}$	8.94	4.45	-
$\tau_{2}^{HH}$	12.95	2.01	2.0 [41]
$\tau_{2}^{\mu }$	22.84	1.57	1.92 [40,42]
$\tau_{2}^{⊥}$	53.28	1.17	-
$\tau_{2}^{OH}$	15.12	1.81	1.95 [43,44,45]

∞ * Bulk water (simulation); ∞ ** Bulk water (experiment).

**Table 3:** The hydrogen bond lifetime of water inside the stacked graphene membrane with different numbers of water molecules.

Number of Water Molecules (*N*_w_)	1698	850	17	3	Bulk Water *	Bulk Water
*τ*_HB_ (ps)	11.0	15.0	18.6	31.7	3.2	~1.0 ** [50,51] 3–10 *** [52,53]
Density of water (H_2_O/Å^3^)	0.037	0.019	3.7 × 10^−4^	6.6 × 10^−7^	0.033	0.033

* our simulation; ** experimental reports in the literature; *** simulation reports in the literature.
